# INPP4B exerts a dual role in gastric cancer progression and prognosis

**DOI:** 10.7150/jca.58397

**Published:** 2021-10-22

**Authors:** Youliang Wu, Xiaodong Wang, Yida Lu, Huizhen Wang, Mingliang Wang, Yexiang You, Xiaoli Su, Dengqun Sun, Yanjun Sun, Yongxiang Li

**Affiliations:** 1 Department of General Surgery, the First Affiliated Hospital of Anhui Medical University, Hefei 230022, People's Republic of China.; 2 Department of Endoscopy Center, the First Affiliated Hospital of Anhui Medical University, Hefei 230022, People's Republic of China.; 3 Department of General Surgery, the Armed Police Corps Hospital of Anhui, Hefei 230041, People's Republic of China.

**Keywords:** gastric cancer, INPP4B, tumour suppressor gene, oncogene, prognostic biomarker

## Abstract

Inositol polyphosphate 4-phosphatase type II (INPP4B) negatively regulates PI3K-Akt signalling and plays diverse roles in different types of cancer, but its role in gastric cancer (GC) is still unknown. Our study aimed to investigate the function and clinical relevance of INPP4B in GC. INPP4B expression was detected in GC tissues and nontumour tissues. The effect of *INPP4B* on the phenotypic changes of AGS and BGC-823 cells was investigated *in vitro*. The activation of serum and glucocorticoid-regulated kinase 3 (SGK3) and AKT were used to evaluate the specific mechanistic function of *INPP4B* in GC cells. The messenger RNA (mRNA) and protein expression levels of *INPP4B* were decreased in GC tissues compared with nontumour tissues. INPP4B expression was associated with tumour-node-metastasis (TNM) stage and histopathological differentiation. In addition, high INPP4B expression in GC patients with large tumour size/low-undifferentiated/TNM's III-IV stage was correlated with a poor prognosis but it was correlated with a better prognosis in patients with small tumour size/high-moderate differentiated/TNM's I-II stage patients. In addition, *INPP4B* knockdown inhibited proliferation, clonal formation and migration and promoted cell apoptosis *in vitro*, while *INPP4B* overexpression led to the opposite effects. Mechanistically, we found that *INPP4B* overexpression enhanced the phosphorylation of SGK3 (p-SGK3) in AGS cells, whereas *INPP4B* knockdown enhanced the p-Akt level in BGC823 cells. These findings suggested that the expression of INPP4B in GC is lower than that in normal tissues. Based on stratification survival analysis and *in vitro* cell experiments, *INPP4B* may play dual roles as an oncogene and tumour suppressor gene in different tissue grades and clinical stages.

## Introduction

Gastric cancer (GC) is the fourth most common cancer and the second most lethal malignancy worldwide, resulting in 951,600 novel cases and 723,100 deaths in 2012 worldwide, especially in East Asian countries 1. Due to the lack of effective early biomarkers, most GC patients are already in advanced stages with a poor prognosis when diagnosed. Therefore, it is imperative to identify additional molecular prognostic biomarkers for GC.

The phosphoinositide 3-kinase (PI3K)-Akt pathway is one of the most common dysregulated cancer-associated signalling pathways and it regulates many cellular functions, such as cell proliferation, growth, survival, migration and metabolism; targeting enzymes in this network has become a major goal of cancer drug development 2. Hyperactivation of PI3K-Akt signalling frequently occurs in human cancers including GC, and it leads to an accumulation of phosphatidylinositol (3,4,5)-trisphosphate [PI(3,4,5)P3] or phosphatidyl inositol (3,4)-bisphosphate [PI(3,4)P2], thus increasing the recruitment and activation of protein kinase Akt to the cytoplasmic membrane. It is the key mediator of PI3K signalling to enhance carcinogenesis 3. However, the signalling lipids PI(3,4,5)P3 and PI(3,4)P2 are degraded by PTEN (phosphatase and tensin homolog) and INPP4B (inositol polyphosphate 4-phosphatase type II) 4, 5.

The tumour suppressor gene (TSG) PTEN is frequently deleted or inactivated in human cancers, including GC. It encodes a lipid phosphatase that dephosphorylates the 3-position phosphate from the inositol ring of PtdIns(3,4,5)P3 to form PtdIns(4,5)P2 to antagonize PI3K-Akt signalling 6. INPP4B, another lipid phosphatase, dephosphorylates the 4-position phosphate from the inositol ring of PtdIns(3,4)P2 to form PtdIns(3)P. As both PI(3,4)P2 and PI(3,4,5)P3 are required for Akt recruitment to the plasma membrane and maximal Akt activation, INPP4B was predicted to act as a tumour suppressor by inhibiting PI3K-Akt signalling 7-9. Indeed, increasing amounts of evidence have identified *INPP4B* as a potential TSG. LOH (loss of heterozygosity) at 4q31.21, the chromosomal region containing *INPP4B*, is reported to occur in basal-like breast tumours (55.6%), ovarian cancers (39.8%) and melanomas (21.6%) 10. In addition, INPP4B protein expression is reduced in several cancers, and its low expression is associated with a poor prognosis 11-14. In agreement with these findings, knockdown of *INPP4B* enhanced Akt activation, anchorage-independent growth, and proliferation in melanoma, breast cancer, and prostate cancer cell lines 11, 13, 15. INPP4B, like PTEN, is anticipated to act as a tumour suppressor by antagonizing PI3K-Akt signalling. However, unlike PTEN, the underlying molecular mechanisms by which INPP4B exerts its tumour suppressive function are poorly understood.

More recently, an increasing number of studies have shown that INPP4B may be upregulated frequently and may play an oncogenic role in several types of cancers 16-18. Even for the same tumour, different researchers have different views on the expression and role of *INPP4B* 18, 19. In short, the role of *INPP4B* in oncogenesis and development remains controversial. The contradictory role of *INPP4B* in different tumours or the same tumour may reflect its extremely complex function in tumours. To date, however, the biological function of *INPP4B* in GC remains unexplored. To further understand the potential value of *INPP4B* in GC, detection of its expression level, evaluation of its clinical prognostic significance, and conducting *in vitro* functional studies are very useful.

In our study, we examined the expression of *INPP4B* in GC using qPCR, western blotting, and immunohistochemistry. We analysed correlations of INPP4B expression with clinicopathological factors by chi-square tests. Furthermore, the prognostic roles of INPP4B in GC were analysed using Cox regression and Kaplan-Meier analysis. Moreover, we examined the biological function of *INPP4B* in GC cell proliferation, apoptosis, colony formation and migration *in vitro*.

## Materials and Methods

### Ethics approval and consent to participate

This study obtained approval from the Institute Research Ethics Committee of the First Affiliated Hospital of Anhui Medical University, and written informed consent was obtained from all the patients enrolled in this study.

### Patients and tissue specimens

In our study, to construct a tissue microarray (TMA), a total of 178 GC and 41 randomly selected normal gastric tissues were gathered from the First Affiliated Hospital of Anhui Medical University of Anhui (Hefei, China) from December 2006 to September 2008. All patients were followed up to December 2014 according to the following rules: regular follow-up every 3 months in the first 2 years and every 6 months in the following years.

Immunohistochemical staining and all of the patients' pathological features were confirmed by two experienced pathologists. Tumour node metastasis (TNM) staging was classified based on the 2010 7th edition of the American Joint Committee on Cancer (AJCC) TNM classification criteria. The detailed clinicopathologic parameters are described in Table 1.

Additionally, thirty-seven matched fresh GC tissues and adjacent nontumour tissue samples were collected and immediately frozen in liquid nitrogen and stored at -80°C from May to August 2019 until use for qPCR and western blotting analysis to explore the differential expression of *INPP4B* mRNA and protein between the GC and adjacent nontumour tissues. All diagnoses were histopathologically confirmed. The inclusion criteria were as follows: 1) histologically confirmed gastric cancer; 2) no preoperative anticancer treatment was given; 3) radical resection of primary lesion; 4) complete clinical information; 5) no other organ or system severe disorders or malignancies. Patients who did not meet the above criteria were excluded.

### RNA preparation, reverse transcription and real-time qPCR

Total RNA was isolated from fresh tissues using TRIzol Reagent (Invitrogen), and reverse transcription (RT) was performed to obtain first-strand cDNA using ReverTra Ace qPCR RT Master Mix (Toyobo) according to the protocol supplied by the manufacturer. The PCR primers were as follows: *INPP4B*, forward primer 5′-ACGCAGGAAAGTCAGGCTAA-3′, reverse primer 5′-TGCCAGGTAACACCATTTCTT-3′; GAPDH was applied as the internal control: forward primer 5′-ATCAAGAAGGTGGTGAAGCAGG-3′, reverse primer 5′-CGTCAAAGGTGGAGGAGTGG-3′. qPCR was carried out using an ABI Prism 7900 HT Sequence Detection System (Applied Biosystems, USA) in the presence of SYBR-Green mix (Toyobo, Japan). Amplification was carried out under the following conditions: denaturation program (95°C for 5 min), followed by an amplification and quantification program for 40 cycles (95°C for 15 s and 60°C for 45 s). Each sample was tested in triplicate. The relative expression of mRNA was calculated using the 2^-ΔΔCT^ method.

### Protein extraction and western blotting

Total proteins were extracted from fresh tissues and cell lines with RIPA lysis buffer containing a cocktail of protease inhibitors (Beyotime Institute of Biotechnology). The protein samples were then separated by 10% SDS-PAGE and electrotransferred to nitrocellulose membranes (Millipore). After blocking with 5% nonfat milk dilution with TBST for 1 h at room temperature, the membrane was incubated with primary antibodies against INPP4B, p-SGK3^T320^, SGK3, p-AKT^T308^, and AKT at 4°C overnight. After washing with TBST, the membranes were incubated with the secondary antibody (Cell Signaling Technology) at a dilution of 1:3000 at room temperature for 60 min. The membranes were then washed with TBST, and the bound antibodies were detected with an enhanced chemiluminescence system. Anti-GAPDH antibody (1:3000; Vazyme) was used as a loading control.

### Immunohistochemistry (IHC)

We provided the tissue samples, and tissue microarray (TMA) manufacturing was done by Shanghai Biochip Company, Ltd., Shanghai, China. Briefly, the immunohistochemistry (IHC) process consisted of the following steps: baking, deparaffinization, antigen retrieval, quenching the endogenous peroxidase activity, blocking nonspecific staining, and incubation with the primary antibody (INPP4B, 1:50, Abcam) and the secondary antibody. The immunoreactivity score (IRS) was defined by “cell staining percentage×staining intensity”. The specimens were defined as negative (IRS =0~2) and positive (IRS=3~9). The detailed immunohistochemical process and staining evaluation were performed as previously described 20.

### Cell culture and lentivirus infection

All cell lines used in this study were obtained from GeneChem (Shanghai, China). All cell lines were cultured in RPMI 1640 supplemented with 10% FBS, penicillin (100 U/ml) and streptomycin (100 μg/ml) and maintained at 37°C in a humidified incubator with 5% CO_2_. To establish the *INPP4B* overexpression AGS cells, AGS cells were infected with *INPP4B* overexpression lentivirus (GV492-INPP4B, OE) and the negative control (GV492, NC, purchased from GeneChem, Shanghai, China). To interfere with *INPP4B* expression and to establish *INPP4B* knockdown BGC-823 cells, the shRNA target sequence for *INPP4B* was 5'-CCATCTGAGTATCCCATCTAT-3′. BGC-823 cells were infected with *INPP4B* shRNA lentivirus (GV248-shINPP4B, KD) and a negative control (GV248, NC, purchased from GeneChem, Shanghai, China). q-PCR and immunoblotting assays were used to estimate the efficiency of the overexpression and knockdown of the *INPP4B* gene.

### Cell proliferation assay and clonogenic assay

Cell proliferation assays and clonogenic assays were used to evaluate the effect of *INPP4B* on the cell growth and proliferation capabilities of GC cells. These assays were performed as previously described 21. For the cell proliferation assay, different lentivirus (OE, KD and NC)-infected cell lines (AGS and BGC-823) were seeded into 96-well plates (approximately 2000 cells/well) in sextuplicate and the cell proliferation assay was performed by MTT (Genview, JT343) according to the manufacturer's instructions. Each experiment was performed at least three times. For the colony formation assay, different lentivirus-infected cell lines were seeded into 6-well plates (approximately 800 cells) in triplicate. These cells were cultured for 14 days, and the medium was replaced every 3 days. Then, the cells were fixed with 4% polyoxymethylene and stained with crystal violet, and the colony numbers (more than 50 cells/colony) were counted using an inverted microscope. The experiments were performed independently in triplicate.

### Apoptosis assay

For apoptosis analysis, different lentivirus-infected cell lines were used with an Annexin V-APC apoptosis detection kit (eBioscience, 88-8007) following the manufacturer's instructions. The apoptosis rate was detected by flow cytometry (BD, FACSCalibur). The detailed apoptotic analysis process was performed as previously described 22.

### Scratch wound-healing assay and Transwell assay

Scratch wound-healing assays and Transwell assays were used to evaluate the cell migration ability and were performed as previously described 14, 23. Briefly, for the scratch wound-healing assay, different lentivirus-infected cell lines were seeded in 96-well plates at approximately 5×10^5^ cells/well. The next day, a scratch tester was used to cross the centre of the cell monolayer. The images were collected by a Celigo instrument, and the migration area of the cells was analysed by its software. For the Transwell assay, different lentivirus-infected cell lines (approximately 1×10^5^ cells/well) in 200 μl serum-free medium were added to the upper chamber of 24-well plates with 8-μm pores, and then 600 μl RPMI-1640 containing 30% FBS was added to the lower chamber. After 24 h of incubation, the noninvading cells were removed by cotton swabs, and the invading cells were fixed with 4% paraformaldehyde for 30 min and stained with 0.5% crystal violet solution. Several random fields of low magnification (×100) and high magnification (×200) were photographed under a microscope. The migratory cell counts were performed using 200× magnification.

### Statistical analysis

All statistical analyses were carried out with SPSS 16.0 software (SPSS Inc., Chicago, IL) and GraphPad Prism 5. Data are expressed as the mean ± SEM. Student's* t*-test and one-way analysis of variance (ANOVA) were used to evaluate the statistical significance of differences between two groups and multiple groups. The relationships between INPP4B expression and the clinicopathologic characteristics were analysed by chi-square tests. Overall survival (OS) was assessed by the Kaplan-Meier method, and statistical significance was analysed using the log-rank test. Cox regression analysis was used to estimate the independent risk factors for OS in GC patients after surgery. P<0.05 indicates statistical significance.

## Results

### Expression of INPP4B mRNA and protein in GC tissues

**Figure 1:**
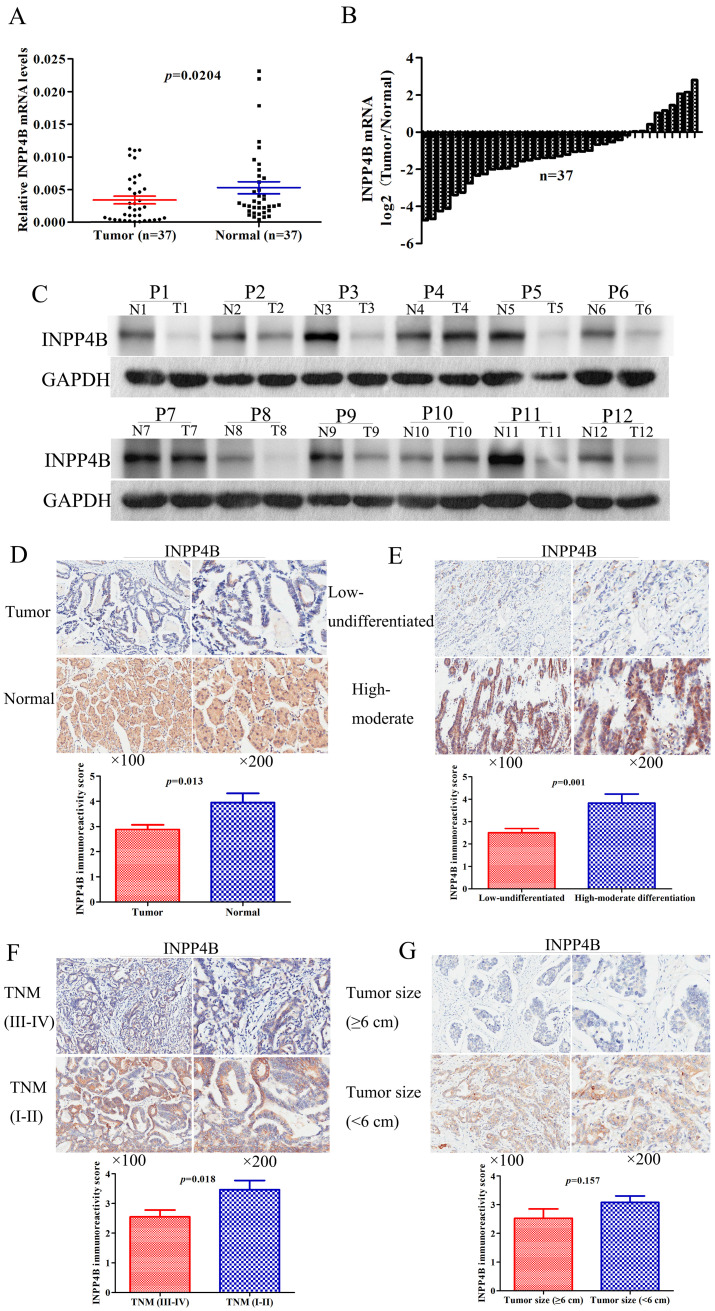
Low expression of *INPP4B* mRNA and protein in GC samples. (A) Scatter plots of the relative expression of *INPP4B* mRNA in tumour tissues and normal controls. (B) Bar plots of *INPP4B* mRNA expression in GC tissues compared with normal controls. (C) The protein expression level of INPP4B was analysed by western blot assay. Representative protein expression level of INPP4B in 12 pairs of tumour samples (T) and corresponding normal controls (N). GAPDH was used as an endogenous control. Immunohistochemistry analysis of INPP4B protein expression in GC and random normal controls. (D) Representative images and immunoreactivity scores of INPP4B in tumour tissues (T) and normal tissues (N). (E) Representative images and immunoreactivity scores of INPP4B in low-undifferentiated and high-moderate differentiation. (F) Representative images and immunoreactivity scores of INPP4B in TNM early stage (I-II) and TNM advanced stage (III-IV); (G) Representative images and immunoreactivity scores of INPP4B in small tumour size (< 6 cm) and large tumour size (≥ 6 cm).

The expression level of INPP4B in tumours is controversial 24, 25. The INPP4B expression pattern in GC is still unknown. qPCR and western blotting were used to investigate *INPP4B* mRNA and protein expression levels in paired fresh frozen tumour-normal tissue specimens. We found that the mean expression level of *INPP4B* mRNA in GC tissues was significantly lower than that in matched controls (P=0.0204, Figure 1A); moreover, 75.68% (28/37) of the tumour tissues had a lower level of *INPP4B* mRNA than the matched controls (Figure 1B). In addition, the expression of INPP4B protein in tumour tissues was decreased compared with that in normal controls (Figure 1C). Taken together, these results suggest that the mRNA and protein expression of *INPP4B* in GC tissues is lower than that in normal controls.

To determine the clinical significance of INPP4B in GC, we analysed the expression of INPP4B in the TMA by immunohistochemistry. We found that the positive immunohistochemical staining was mainly localized in the cytoplasm and that it exhibited a significant difference: the positive rate was significantly lower in the GC tissues than in normal controls [72/178 (40.45%) vs. 31/41 (75.61%), P < 0.001]. Representative images of INPP4B immunohistochemical staining in GC tissue and normal controls are shown in Figure 1D. In summary, our results showed that INPP4B is expressed at low levels in GC and it is mainly located in the cytoplasm.

### Associations of INPP4B expression with clinicopathological parameters

To further elucidate the roles of INPP4B in the pathogenesis of GC, we analysed the relationships between the expression of INPP4B and clinicopathological parameters. The tumour TNM stage was classified as early stage (I-II) and advanced stage (III-IV). The histopathological differentiation was divided into high-moderate and low-undifferentiated. The lesional lymph node stages were classified as lymph node-negative (No) and lymph node-positive (Yes). As shown in Table 1, INPP4B expression was significantly correlated with TNM tumour stage (I-II vs. III-IV, P=0.033) and histopathological differentiation (high-moderate vs. low-undifferentiated, P=0.019) and weakly related to tumour size (<6 cm vs. ≥6 cm, P=0.08) but not related to any other clinical parameters. The IRS in the tumour tissues was decreased compared with that in the normal controls (Figure 1D). Compared to high-moderate differentiation, TNM early stage (I-II) and small tumour size (<6 cm), the IRS of INPP4B was significantly decreased in low-undifferentiated and TNM advanced stage (III-IV, Figure 1E, 1F), while the IRS of INPP4B was slightly decreased in large tumour size (≥6 cm, Figure 1G), not reaching statistical significance. Collectively, our results indicate that the expression level of INPP4B may be related to tumour size, histopathological differentiation and TNM tumour stage.

### Survival analysis

**Figure 2:**
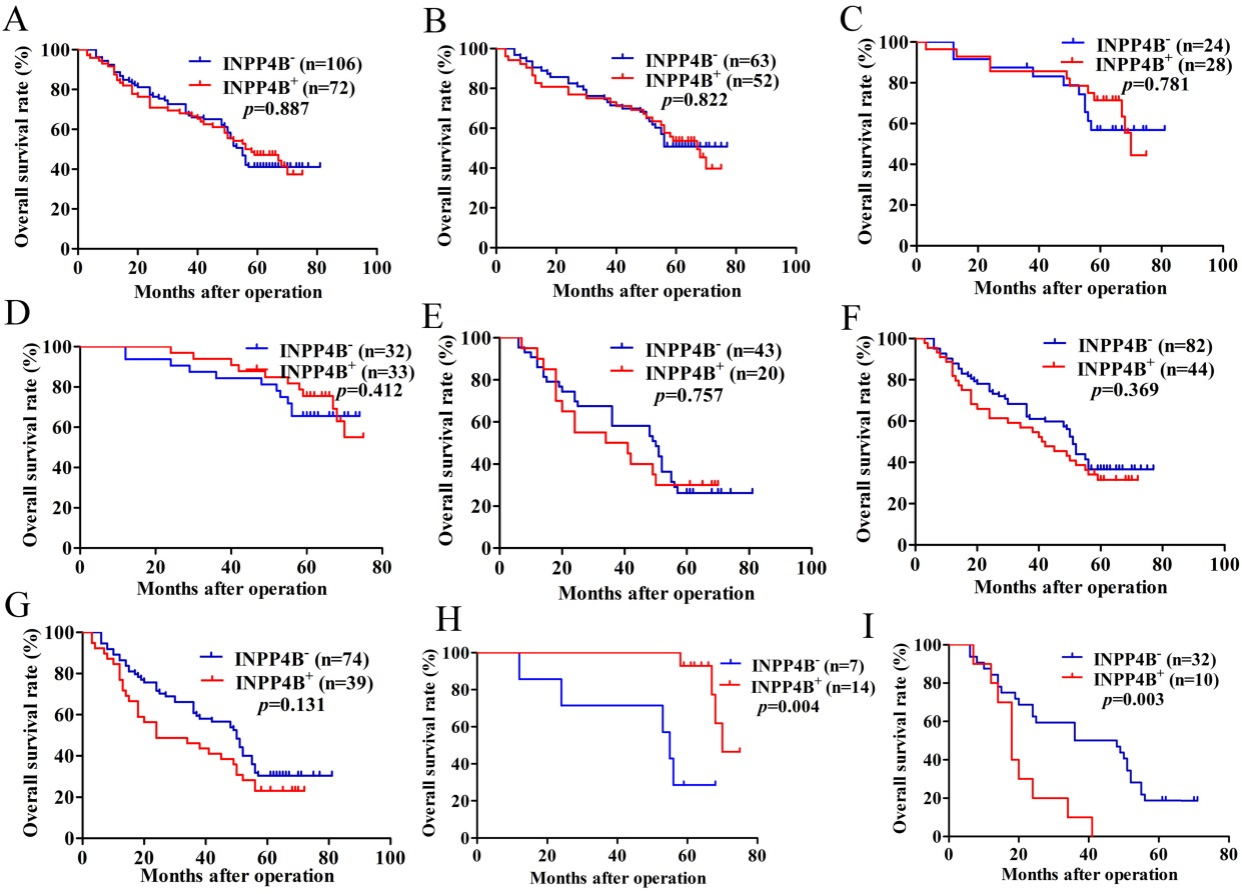
Kaplan-Meier survival analysis with the log-rank test for the OS of 178 GC patients. (A) The OS of GC patients with INPP4B^-^ and INPP4B^+^. (B) The OS of GC patients with small tumour sizes (< 6 cm) who were INPP4B^-^ and INPP4B^+^. (C) The OS of GC patients with high-moderate differentiation with INPP4B^-^ and INPP4B^+^. (D) The OS of GC patients in TNM early stage (I-II) with INPP4B^-^ and INPP4B^+^. (E) The OS of GC patients with a large tumour size (≥6 cm) who were INPP4B^-^ and INPP4B^+^. (F) The OS of GC patients in low undifferentiated grade with INPP4B^-^ and INPP4B^+^. (G) The OS of GC patients in TNM advanced stage (III-IV) with INPP4B^-^ and INPP4B^+^. (H) The OS of GC patients in small tumour size (< 6cm)/high-moderate differentiation/TNM early stage (I-II) with INPP4B^-^ and INPP4B^+^. (I) The OS of GC patients in large tumour size (≥ 6cm)/low-undifferentiated/TNM advanced stage (III-IV) with INPP4B^-^ and INPP4B^+^.

Based on the above results, we surmised that INPP4B might be related to the GC prognosis. Kaplan-Meier analysis and log-rank tests were used to evaluate the prognostic value of INPP4B in GC patients. However, when we did not stratify the relationship between INPP4B and GC prognosis, we found that INPP4B expression was not associated with overall survival (OS). There was no significant difference in OS between GC patients with INPP4B^-^ expression (median 55 months) and those with INPP4B^+^ (median 56 months, P=0.887, Figure 2A). Because TNM stage contains information on the depth of invasion and lymph node metastasis, we did not add these two factors in the stratification analysis of tumour size, differentiation and TNM stage. When we stratified the relationship between INPP4B and the prognosis of GC in terms of tumour size, differentiation, and TNM staging, we obtained the following interesting findings.

In the small tumour size (<6 cm) group, high-moderate differentiation group and TNM early stage (I-II) group, INPP4B^+^ GC patients seems to have a longer OS than INPP4B^-^ GC patients (Figure 2B, 2C, 2D, respectively). However, in the large tumour size (≥6 cm) group, low-undifferentiated group and TNM advanced stage group (III-IV), GC patients with INPP4B^+^ seemed to have a shorter OS than patients with INPP4B^-^ (Figure 2E, 2F, 2G, respectively). These results did not reach statistical significance, which may be due to the small number of cases. It can be inferred from the existing results that INPP4B may act as a TSG in the well differentiated tissue grade and early clinical stage, and as an oncogene in the worse differentiated tissue grade and advanced clinical stage. To eliminate the interaction between these factors, we further stratified the relationship between these factors and the clinical prognosis. We found that small tumour size (<6 cm)/high-moderate differentiation/TNM early stage (I-II) patients with INPP4B^+^ had a more favourable prognosis than patients with INPP4B^-^ (Figure 2H), whereas large tumour size (≥ 6cm)/low-undifferentiated/TNM advanced stage (III-IV) patients with INPP4B^+^ had a worse prognosis than patients with INPP4B^-^ (Figure 2I). Taken together, these results indicate that *INPP4B* plays different roles in GC progression in different tissue grades and clinical stages.

Finally, we further assessed the predictive values of INPP4B in GC prognosis by Cox regression analysis. When we did not stratify the relationship between INPP4B and GC prognosis, multivariate analyses only revealed that the depth of invasion, lymph node metastasis and differentiation were independent predictor factors of GC patients (Table 2). When we stratified the relationship between INPP4B and GC prognosis, univariate analysis revealed that INPP4B was a significant predictor of a good outcome for GC patients in the small tumour size (<6 cm)/high-moderate differentiation/TNM early stage (I-II) group (Table 3), but it was a significant predictor of a poor outcome in the large tumour size (≥ 6cm)/low-undifferentiated/TNM advanced stage (III-IV) group (Table 4). Taken together, our results indicate that *INPP4B* plays a contradictory predictive role in GC after stratification by tissue grade and clinical stage.

### INPP4B regulates GC cells proliferation *in vitro*

**Figure 3:**
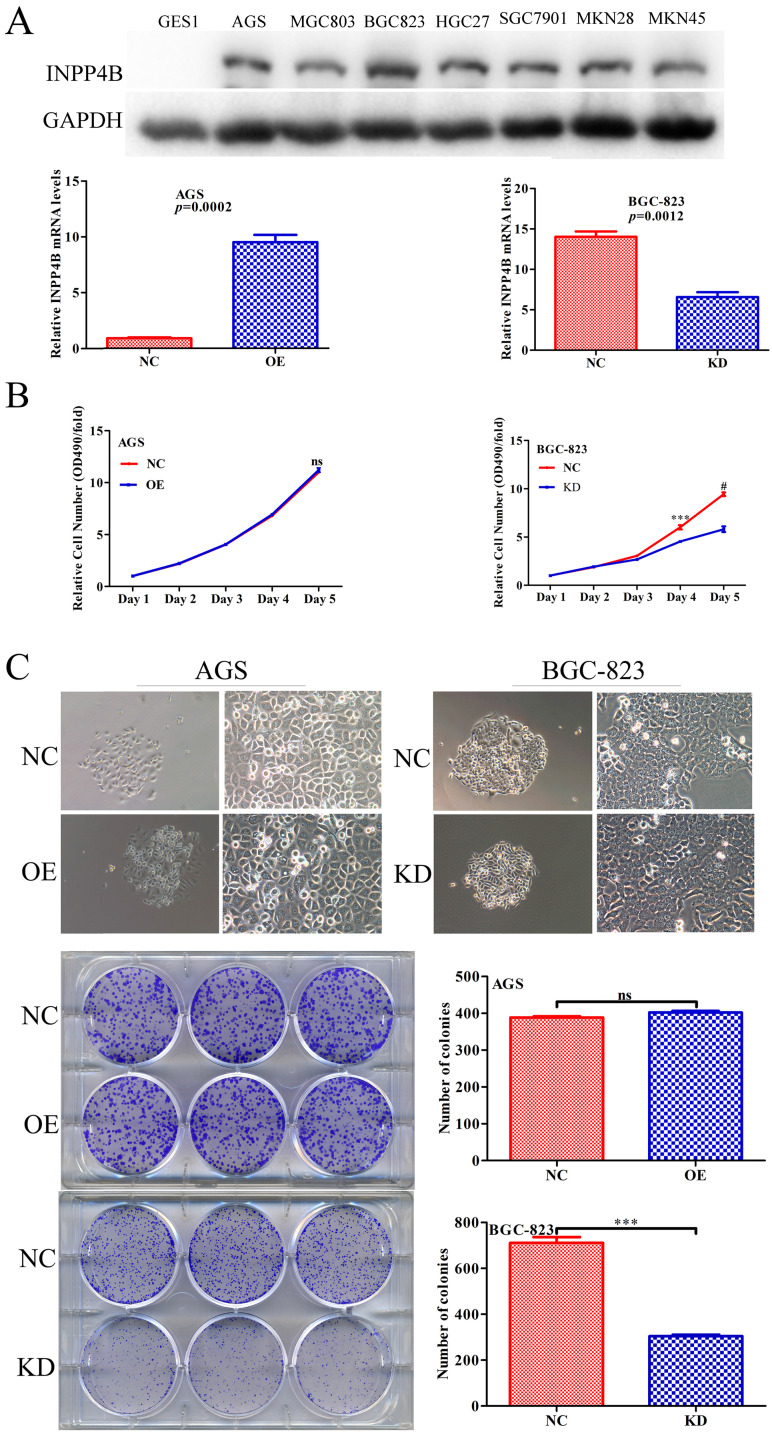
*INPP4B* controls GC cell growth *in vitro*. (A) The protein and mRNA expression of *INPP4B* in GC cell lines before and after infection with different lentiviruses. (B) Proliferation curve for AGS cells and BGC-823 cells with *INPP4B* overexpression (OE), knockdown (KD) and negative control (NC). (C) Colony formation of AGS cells and BGC-823 cells with *INPP4B* overexpression (OE), knockdown (KD) and negative control (NC). Each experiment was repeated three times. Values are expressed as the means ± SEM. ***, P<0.001; #, P<0.0001; ns, not significant.

Given that *INPP4B* has dual functions in GC clinical prognosis, we inferred that *INPP4B* might have dual functions *in vitro*. We chose AGS cells for overexpression and BGC-823 cells for knockdown because *INPP4B* was highly expressed in BGC-823 cells, while AGS cells and other GC cell lines were relatively low expressed (Figure 3A). To verify our hypothesis, we established AGS and BGC-823 cells stably overexpressing, silenced and negative control *INPP4B* by infection with different lentiviruses. Then, we performed MTT and colonigenic assays to examine the effects of *INPP4B* on the growth and proliferation of AGS and BGC-823 cells. As a result, *INPP4B* knockdown significantly inhibited BGC-823 cell proliferation and colony formation, while *INPP4B* overexpression weakly promoted AGS cell proliferation and colony formation (Figure 3B and 3C), which did not reach a statistically significant level. In summary, our findings suggest that the inhibitory effect of *INPP4B* knockdown on GC cell proliferation and colony formation is stronger than the promoting effect of *INPP4B* overexpression.

### INPP4B inhibits GC cells apoptosis *in vitro*

**Figure 4:**
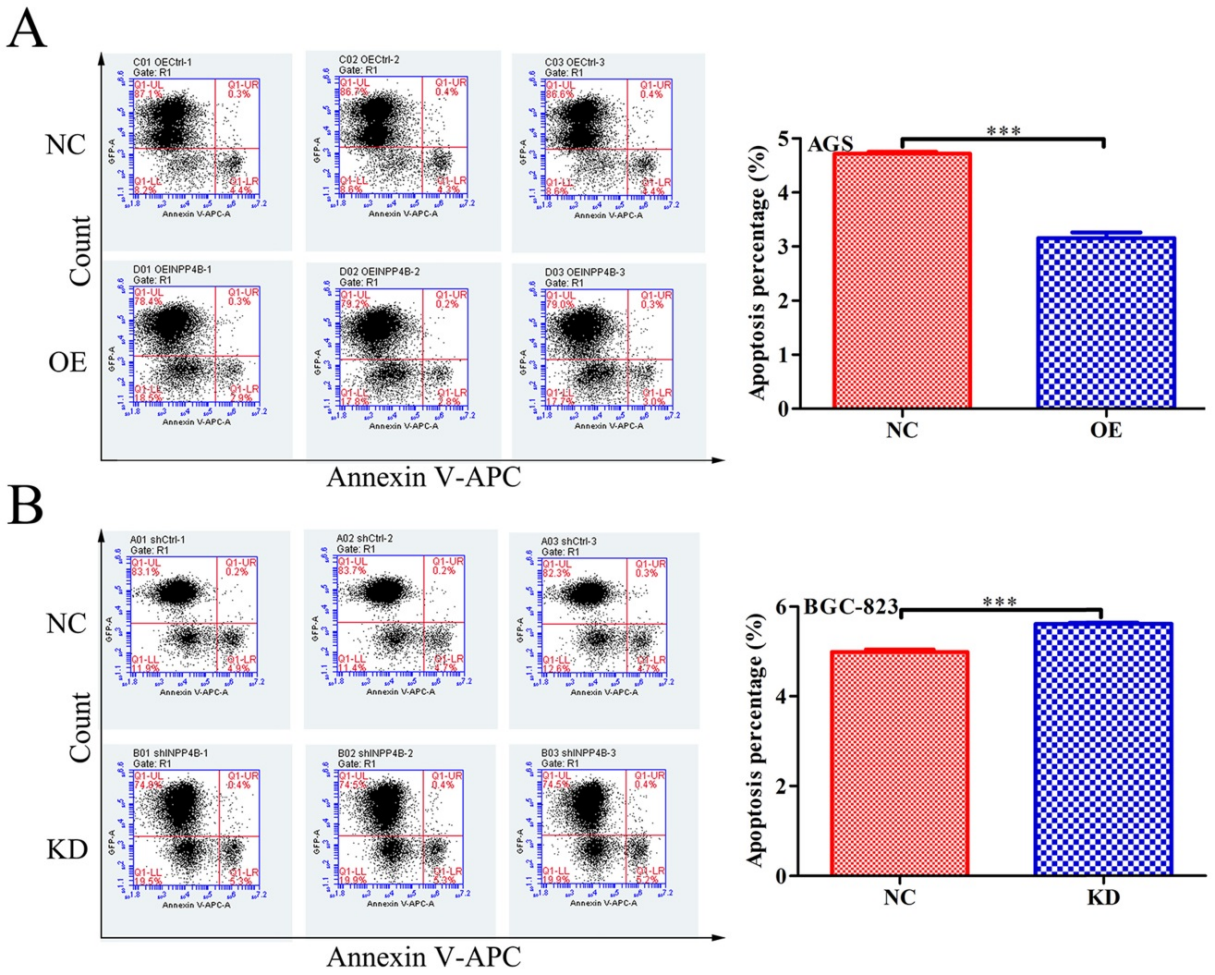
*INPP4B* controls GC apoptosis *in vitro*. (A) Overexpression of *INPP4B* significantly inhibits AGS apoptosis. (B) Knockdown of *INPP4B* significantly induces BGC-823 apoptosis. ***, P<0.001.

To further verify our hypothesis, we next investigated the effect of *INPP4B* on apoptosis in GC cells using flow cytometry. Our date showed that *INPP4B* overexpression significantly reduced the apoptosis rate of AGS cells (Figure 4A), and *INPP4B* knockdown significantly increased the apoptosis rate of BGC-823 cells (Figure 4B). These date indicate that *INPP4B* inhibits the apoptosis of GC cells.

### INPP4B promotes GC cells migration *in vitro*

**Figure 5:**
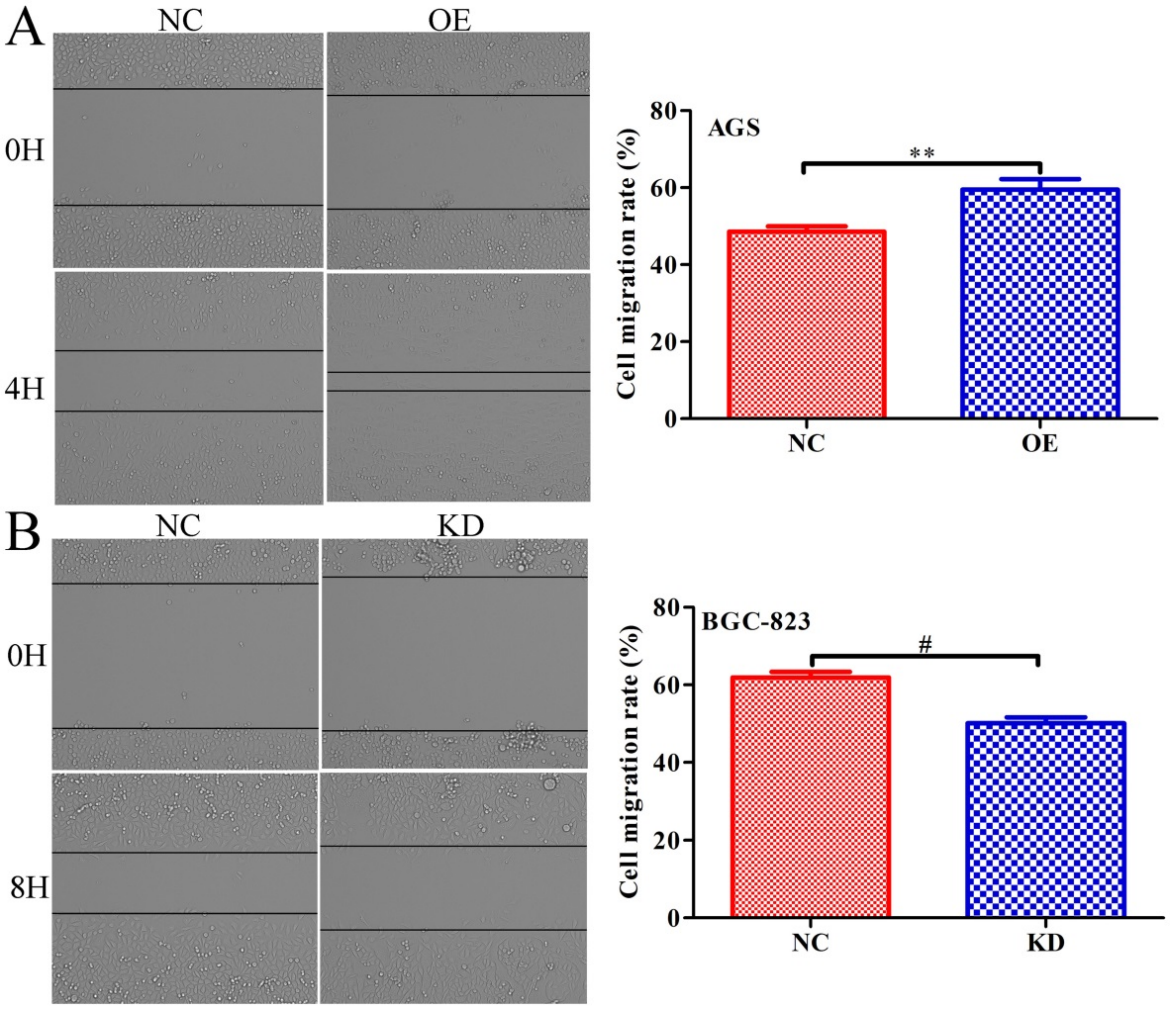
*INPP4B* controls GC cell migration as demonstrated by a scratch wound-healing assay. (A). Overexpression of *INPP4B* significantly promotes AGS cell migration. (B). Knockdown of *INPP4B* significantly inhibits BGC-823 cell migration. **, P<0.01; #, P<0.0001.

**Figure 6:**
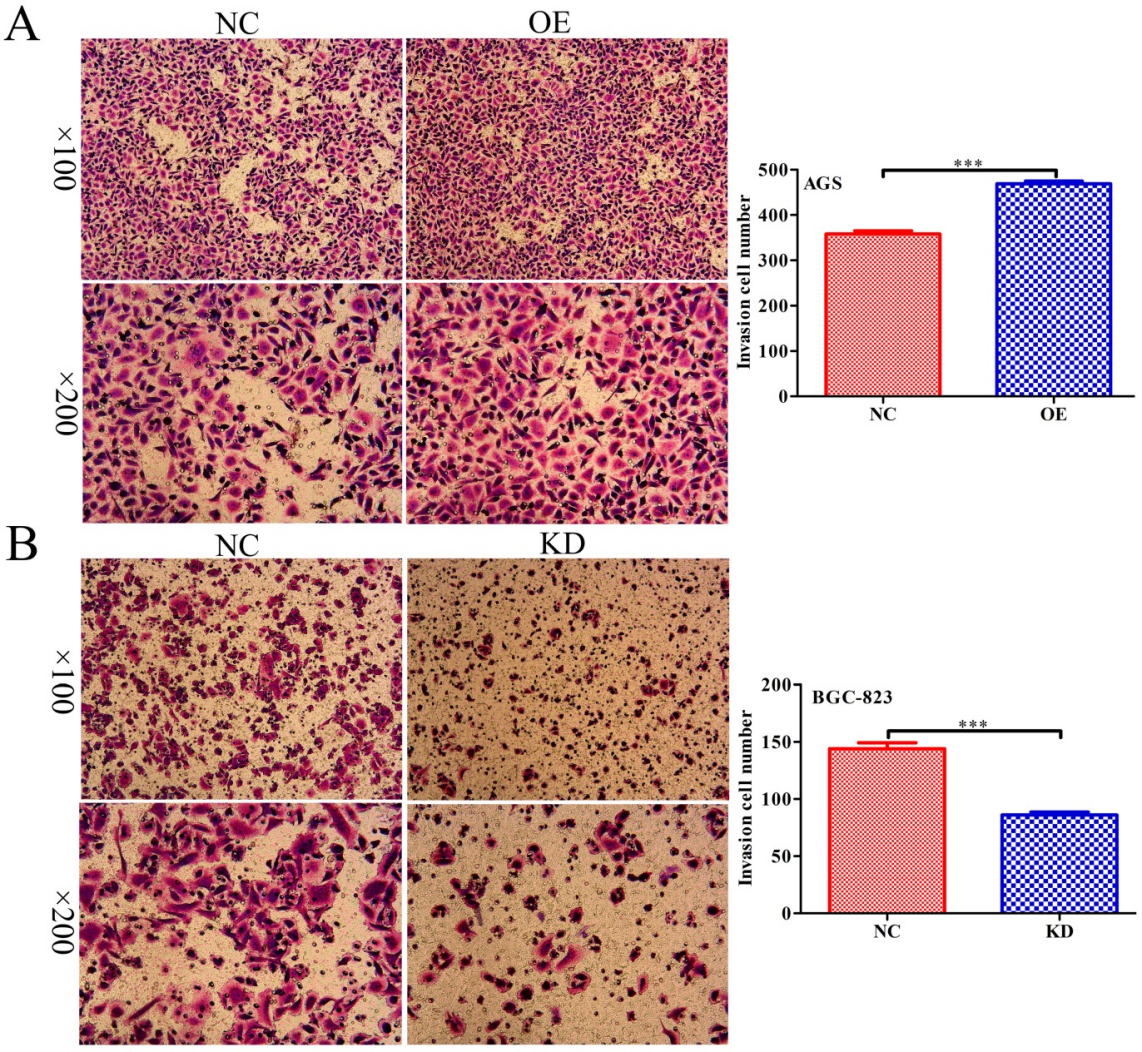
*INPP4B* regulates GC cell migration as demonstrated by a Transwell assay. *INPP4B* overexpression enhances AGS cell migration. (B). *INPP4B* knockdown inhibits BGC-823 cell migration. ***, P<0.001.

To further explore whether *INPP4B* affects the migration of GC cells, scratch wound healing and Transwell assays were performed. Scratch wound-healing assays and Transwell assays revealed that *INPP4B* overexpression notably increased AGS cell migration (Figure 5A and Figure 6A), while *INPP4B* knockdown significantly reduced BGC-823 cell migration (Figure 5B and Figure 6B). Taken together, our findings indicate that *INPP4B* promotes GC cell migration *in vitro*.

### INPP4B mediates SGK3 and Akt activation in GC cells

**Figure 7:**
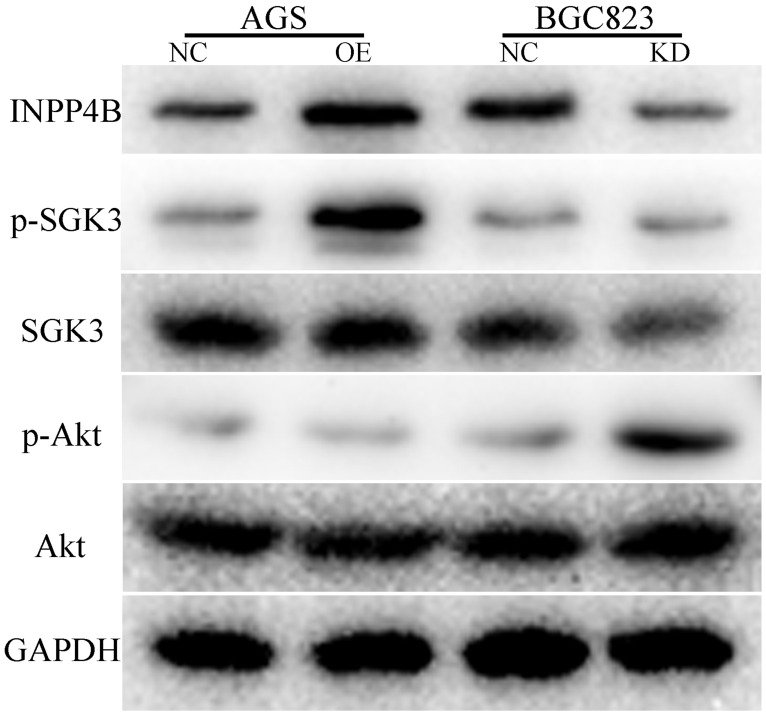
INPP4B affects the biological functions of AGS and BGC823 cells through different signalling pathways. INPP4B overexpression enhanced the phosphorylation of SGK3 (p-SGK3) in AGS cells; whereas INPP4B knockdown enhanced the p-Akt level in BGC823 cells.

To explore the possible mechanism by which I*NPP4B* promotes GC cell progression, we used WB to detect the changes in some important downstream proteins of INPP4B, such as p-SGK3(Thr320), SGK3, p-Akt(Ser473) and AKT, in AGS-overexpressing and BGC-823-knockdown cell lines. Our results showed that INPP4B overexpression enhanced the phosphorylation of SGK3 (p-SGK3), but did not affect the phosphorylation of Akt (p-Akt) in AGS cells; whereas INPP4B knockdown enhanced the p-Akt level but did not enhance the p-SGK3 level in BGC823 cells (Figure 7). Taken together, these results suggest that *INPP4B* may affect the biological functions of AGS and BGC823 cells through different signalling pathways.

## Discussion

GC is one of the major threats to human health and it has a poor prognosis. Despite great advances in diagnosis and treatment methods for GC, the long-term survival of patients remains unsatisfactory. Currently, the prognostic system routinely employed for tumour management is primarily dependent on the American Joint Committee on Cancer (AJCC) TNM staging system 24. However, we cannot predict the clinical outcome of patients after surgery depending on clinical parameters alone, because the biological aggressiveness of each individual disease is characterized by its potential for metastasis and its resistance to anticancer therapy. Therefore, identifying significant molecular biological prognostic factors may help achieve a more accurate prediction of the clinical outcome and may also reveal novel predictive factors and therapeutic targets 25.

The PI3K-Akt signalling pathway was confirmed to be a typical pathway that triggers a cascade of responses, including cell growth, metastasis, EMT, angiogenesis, and the development of chemoresistance in a wide range of tumour types 26-28. Deregulation of the PI3K-Akt signalling pathway is associated with numerous human cancers, including GC 29-31. INPP4B negatively regulates the PI3K-Akt signalling pathway, which is expressed at low levels and has a tumour-suppressive role in several types of human malignancies 13-15. However, unexpected findings from recent reports indicated that *INPP4B* is highly expressed in some malignancies and plays a role as an oncogene 16-18. Evidence accumulated from basic and clinical studies suggests that *INPP4B* may play a very controversial role in cancer progression.

There was only one previous report on *INPP4B* in GC. Choi et al. reported that they found a frameshift mutation of the *INPP4B* gene in GC, which created a premature stop codon, leading to functional inactivation of the protein 32. However, the expression level and clinical significance of *INPP4B* in GC are unknown. In our study, we analysed the expression of *INPP4B* in GC and its prognostic implications. The mRNA expression level of INPP4B was consistent with the protein expression level, and the expression level was low in GC tissues compared with normal tissues. These results indicate that the INPP4B protein level is low in GC tumour tissues, which may be at least partially caused by decreased transcription of the *INPP4B* gene.

As shown in Table 1 and Figure 1, we found an interesting phenomenon in which the expression of *INPP4B* in small tumour size (< 6cm), high-moderate histopathological differentiation and TNM early stage (I-II) was increased compared with that in large tumour size (≥ 6cm), low-undifferentiated histopathological differentiation and TNM advanced stage (III-IV). However, survival analysis and Cox proportional hazards model analysis found that when we did not stratify the relationship between INPP4B and GC prognosis, *INPP4B* expression was independent of OS. When we stratified the relationship between INPP4B and the prognosis of GC in terms of tumour size, differentiation, and TNM staging, we found that GC patients with high expression of *INPP4B* had a better prognosis in the well differentiated tissue grade and early clinical stage but had a poor prognosis in the worse tissue grade and advanced clinical stage, which indicated a contradictory role. These findings demonstrated the dual function of *INPP4B* in different tissue grades and clinical stages.

Previous studies on primary non-metastatic and metastatic colorectal cancer stem-like cells (CR-CSLCs) have found that *INPP4B* is expressed at low levels in non-metastatic CR-CSLCs and at high levels in metastatic CR-CSLCs, plays a tumour suppressor role in non-metastatic CR-CSLCs cells and plays an oncogenic role in metastatic CR-CSLCs, depending on different molecular mechanisms 4. This has only been demonstrated at the cellular level *in vitro* so far, but we found for the first time that *INPP4B* plays a dual role in different tissue grades and clinical stages of the same type of tumour. We cannot explain this phenomenon fully, but it is likely that *INPP4B* regulates different molecular signalling pathways in different tissue grades and clinical stages of GC. These results suggested that *INPP4B* was likely to play important roles in the progression of GC. However, these results need to be validated by further study of a larger cohort of GC patients. Next, we further elucidated whether *INPP4B* has dual functions in GC cells *in vitro*.

Previous studies on the role of *INPP4B* in tumour cells have shown that *INPP4B* plays different roles in different tumour cells. Knockdown of *INPP4B* in breast cancer cells results in enhanced Akt activation, cell proliferation, anchorage-independent growth and motility 10, 15. Overexpression of *INPP4B* in prostate cancer cells results in suppressed migration, invasion and angiogenesis 33. Zhang et al. found that IRF2 serves as an oncogenic protein in human AML by promoting the expression of *INPP4B* to promote the growth of AML cells 34. Guo et al. found that silencing *INPP4B* blocks the activation of Akt and serum- and glucocorticoid-regulated kinase 3 (SGK3), inhibits colon cancer cell proliferation and delays colon cancer xenograft growth 18. These results indicate that *INPP4B* functions as an oncogene or TSG in different types of cancer cells.

When we tested the basal expression of *INPP4B* in GC cell lines by western blot, it was found that INPP4B was expressed in all gastric cancer cells but not in normal (GES1) cells, which was consistent with the role of INPP4B as an oncogene in the phenotype experiment of gastric cancer cells. We chose two GC cell lines (AGS and BGC-823) for the *in vitro* functional assays. Overexpression of *INPP4B* in AGS cells significantly reduced the cell apoptosis rate, increased the cell migration capability, and weakly promoted cell proliferation and colony formation; knockdown of *INPP4B* in BGC-823 cells significantly increased the apoptosis rate, decreased the cell migration capability, and decreased proliferation and colony formation. All of the above phenotypic experiments support the hypothesis that *INPP4B* plays an oncogenic in gastric cancer cells.

However, we also used WB to explore the possible mechanism by which *INPP4B* promotes GC cell progression. We found that INPP4B may affect the biological functions of AGS and BGC823 cells through different signalling pathways. What makes us wonder is that INPP4B knockdown in BGC823 cells can lead to the activation of p-Akt, a typical role of tumour suppressors, but the phenotypic experiment suggested it functioned like an oncogene, which seems to be contradictory. This may be because INPP4B in BGC823 cells can affect other unknown signalling pathways in addition to p-Akt activation. According to these results, *INPP4B* is more likely to act as an oncogene in GC cells. However, the mechanism by which *INPP4B* acts as an oncogene in GC cells is not fully understood. Therefore, more biological experiments and mechanistic studies should be carried out to verify this finding using more cell lines.

In conclusion, our study has demonstrated for the first time that *INPP4B* is expressed at low levels in Chinese GC tissues and it plays a dual role in the prognosis of GC patients. Our results show that GC patients with high expression of INPP4B have a better prognosis in the well-differentiated tissue grade and early clinical stage group, but patients in the worse-differentiated tissue grade and advanced clinical stage group have poor prognosis, indicating that INPP4B plays a contradictory role. Additionally, INPP4B may act as an oncoprotein in GC cells. Using *in vitro* analyses, we found that knockdown of *INPP4B* in BGC-823 cells could increase the apoptosis rate, decrease cell migration capability, and reduce proliferation and colony formation, while overexpression of *INPP4B* in AGS cells had the opposite effect, suggesting that *INPP4B* is an oncogene in GC cells. However, due to the paradoxical roles of *INPP4B* in the prognosis of GC patients and other cancers, further biological experiments and mechanistic studies of *INPP4B* in GC are necessary. Moreover, understanding the molecular mechanisms underlying the role of *INPP4B* in the development of GC will provide a novel treatment approach for GC.

## Figures and Tables

**Table 1:** Relationship between INPP4B expression and clinicopathological variables (n=178).

Clinicopathological variables	Total	INPP4B expression		χ2	P value
		positive	negative		
Gender				0.236	0.627
Male	132	52	80		
Female	46	20	26		
Age (y)				0.298	0.585
<61	86	33	53		
≥61	92	39	53		
Tumour size (cm)				3.066	0.08
<6	115	52	63		
≥6	63	20	43		
Differentiation				5.473	**0.019**
High/moderate	52	28	24		
Low/undifferentiated	126	44	82		
Depth of invasion				0.125	0.724
T1/T2	30	13	17		
T3/T4	148	59	89		
Lymph node metastasis				2.18	0.14
Yes	127	47	80		
No	51	25	26		
TNM				4.527	**0.033**
I/II	65	33	32		
III/IV	113	39	74		
Location				0.587	0.746
Upper	86	37	49		
Middle	39	14	25		
Lower	53	21	32		

Note: TNM, tumour-node-metastasis; P values were detected by the Pearson Chi-square test; P<0.05 was defined statistically significant and was given in bold.

**Table 2:** Univariate and multivariate analysis of the correlation between clinicopathological parameters and prognostic significance of GC patients (n=178).

Variables	Univariate analysis	*p* value	Multivariate analysis	P value
	HR(95%CI)		HR(95%CI)	
Gender (male vs. female)	0.778(0.489-1.238)	0.289		NA
Age (y) (<61 vs. ≥61)	1.053(0.715-1.550)	0.794		NA
Tumour diameter (cm) (<6 vs. ≥6)	0.545(0.368-0.807)	**0.002**	0.793(0.531-1.184)	0.257
Differentiation (high/moderate vs. low/undifferentiated)	2.207(1.364-3.570)	**0.001**	2.147(1.311-3.514)	**0.002**
Location (upper vs middle vs. lower)	0.916(0.732-1.145)	0.439		NA
Depth of invision (T1/TI vs. T3/T4)	6.083(2.473-14.965)	**<0.0001**	4.027(1.507-10.763)	**0.005**
Lymph node metastasis (yes vs. no)	3.880(2.203-6.833)	**<0.0001**	2.718(1.313-5.623)	**0.007**
TNM stages (I/II vs. III/IV)	3.390(2.109-5.449)	**<0.0001**	1.134(0.585-2.197)	0.71
INPP4B (positive vs. negative)	0.972(0.655-1.443)	0.888		NA

Note: Variables with P values more then 0.05 in the univariate models were not adapted (NA) in the multivariate analysis. P<0.05 was defined statistically significant and was given in bold. CI: confidence interval. HR: Hazard ratio.

**Table 3:** Univariate analysis of GC patients with small tumour size (<6cm)/high-moderate differentiation/TNM early stage (I-II) (n=21).

Variables	Univariate analysis	P value
	HR(95%CI)	
Gender (male vs. female)	5.223(0.984-27.734)	0.052
Age (y) (<61 vs. ≥61)	0.670(0.179-2.508)	0.552
Location (upper vs middle vs. lower)	0.674(0.226-2.011)	0.479
Depth of invision (T1/TI vs. T3/T4)	5.333(0.660-43.085)	0.116
Lymph node metastasis (yes vs. no)	2.864(0.710-11.555)	0.139
INPP4B (positive vs. negative)	0.154(0.036-0.662)	0.012

Note: P<0.05 was defined statistically significant and was given in bold. CI: confidence interval. HR: Hazard ratio.

**Table 4:** Univariate analysis of GC patients with large tumour size (≥6cm)/low-undifferentiated/ TNM advanced stage (III-IV) (n=42).

Variables	Univariate analysis	P value
	HR(95%CI)	
Gender (male vs. female)	0.729(0.317-1.675)	0.457
Age (y) (<61 vs. ≥61)	1.058(0.549-2.038)	0.867
Location (upper vs middle vs. lower)	0.948(0.642-1.402)	0.79
Depth of invision (T1/TI vs. T3/T4)	0.921(0.124-6.817)	0.936
Lymph node metastasis (yes vs. no)	3.146(0.430-23.048)	0.259
INPP4B (positive vs. negative)	3.219(1.417-7.312)	**0.005**

Note: P<0.05 was defined statistically significant and was given in bold. CI: confidence interval. HR: Hazard ratio.

## Abbreviations

INPP4B: inositol polyphosphate 4-phosphatase type II
GC: gastric cancer
SGK3: serum and glucocorticoid-regulated kinase 3
TNM: tumour-node-metastasis
PTEN: phosphatase and tensin homolog
TSG: tumour suppressor gene
LOH: loss of heterozygosity
TMA: tissue microarray
AJCC: American Joint Committee on Cancer
IHC: immunohistochemistry
IRS: immunoreactivity score
OS: Overall survival
